# Historische Entwicklung in Diagnostik und Therapie bei Präexzitationssyndromen (WPW)

**DOI:** 10.1007/s00399-024-01000-6

**Published:** 2024-03-01

**Authors:** Boris Rudic, Martin Borggrefe

**Affiliations:** grid.411778.c 0000 0001 2162 1728 I. Medizinische Klinik, Universitätsmedizin Mannheim, 68167 Mannheim, Theodor-Kutzer-Ufer 1–3, Deutschland

**Keywords:** Präexzitation, WPW-Syndrom, Akzessorische Leitungsbahn, Radiofrequenzablation, Historie, Pre-excitation, Wolff-Parkinson-White syndrome, Accessory pathway, Radiofrequency ablation, History

## Abstract

Wolff, Parkinson und White beschrieben 1930 das Syndrom, das nach ihnen benannt wurde. Die Mechanismen der supraventrikulären Tachykardien wurden schon durch brillante Interpretation des Oberflächen-EKGs durch Pick und Langendorf erforscht. Wellens und Durrer analysierten invasiv mittels programmierter Stimulation die Rhythmusstörungen beim WPW-Syndrom. In der BRD waren die Arbeitsgruppen um Seipel und Breithardt sowie Neuss und Schlepper aktiv in der Erforschung der Tachykardiemechanismen und der Effekte von Antiarrhythmika. Nach der ersten operativen Durchtrennung einer akzessorischen Leitungsbahn durch Sealy 1967 etablierten sich operativ tätige elektrophysiologische Teams auch in der BRD, u. a. in Hannover und Düsseldorf. Die Gleichstromkatheterablation hielt Einzug in der kurativen Therapie des WPW-Syndroms durch Morady und Scheinman. Wegen der Nebenwirkungen des Barotraumas bei Gleichstromablation wurden alternative Therapiestrategien erforscht. 1987 hielt die Radiofrequenzablation (RF-Ablation) Einzug in die nichtpharmakologische Therapie des WPW-Syndroms und hat sich seither als Therapiestandard von akzessorischen Leitungsbahnen in allen Lokalisationen etabliert.

## Abstract (en)

In 1930, Wolff, Parkinson and White described the syndrome that bears their names. The mechanisms of supraventricular tachycardias were analyzed by brilliant electrocardiography interpretation by Pick and Langendorf. Wellens and Durrer using electrophysiologic studies analyzed the tachycardia mechanism invasively. In Germany the group by Seipel and Breithardt as well as Neuss and Schlepper studied the tachycardia mechanisms and response to antiarrhythmic drugs invasively by electrophysiological studies. Following the first successful interruption of an accessory pathway by Sealy in 1967, surgeons and electrophysiologists cooperated in Germany. Two centers, Hannover and Düsseldorf were established. Direct current (DC) ablation of accessory pathways was introduced by Morady and Scheinman. Because of side effects induced by barotrauma of DC, alternative strategies were studied. In 1987, radiofrequency ablation was introduced and thereafter established as curative therapy of accessory pathways in all locations.

## Die ersten Beschreibungen

In einer gemeinsamen Publikation aus Boston und London beschrieben 1930 Wolff, Parkinson und White [48] das von ihnen benannte Syndrom. Es wurden 11 Patienten in die Publikation eingeschlossen, die kardial gesund waren und unter paroxysmalen Tachykardien und/oder Vorhofflimmern litten. Das EKG zeigte eine verkürzte PQ-Zeit und einen verbreiteten QRS-Komplex. Sie beschrieben auch, dass die T‑Welle entgegen der Depolarisation ausgerichtet war. Unter Belastungsbedingungen kam es zu einer Normalisierung des QRS-Komplexes. Sie beschrieben auch, dass Quinidin die Neigung zu Tachykardien positiv beeinflusste. In dieser Publikation zitieren die Autoren einen Fallbericht von Frank Wilson von 1915 [47], der ein WPW-Syndrom erstmalig in der angloamerikanischen Literatur beschrieb. Von Knorre [43] hat sich die Mühe gemacht und nach dem ersten publizierten EKG eines WPW-Syndroms gesucht. Er konnte zeigen, dass bereits 1909 Hoffmann [15] ein EKG mit einem WPW-Muster publizierte. August Hoffmann (1862–1929) war in Düsseldorf tätig und war lange bevor das EKG eingeführt wurde an Tachykardien interessiert. „Die paroxysmale Tachykardie“ wurde 1900 publiziert [16].

Im Jahr 1932 postulierten Holzmann und Scherf, dass die Antesystolie (Delta-Welle) durch eine vorzeitige Erregung der Herzkammer über eine zusätzliche Leitungsbahn zu Stande kommt. Diese Theorie wurde 10 Jahre später durch Autopsien bestätigt und gilt nun schon lange durch Tausende von chirurgischen oder elektrophysiologischen Unterbrechungen akzessorischer Leitungsbahnen als bewiesen. Dass das Phänomen heute in der Literatur ohne den Namen Holzmann als „Syndrom von Wolff-Parkinson-White“ bekannt ist, mag ihn als Schweizer ärgern, zu ändern ist es leider nicht mehr.

Noch bevor das WPW-Syndrom beschrieben wurde, spekulierte 1914 Mines [28], dass es eine kreisende Erregung geben könnte, die über eine retrograde Erregung über ein exzentrisches Muskelbündel verläuft und über den Vorhof und das normale Reizleitungssystem die Kammer wieder erregt (kreisende Erregung). Pick, Langendorf und Katz untersuchten unzählige EKGs und postulierten lange vor der Einführung der invasiven Elektrophysiologie die Induktionsmechanismen von Tachykardien bei WPW-Syndrom und beschrieben auch „concealed conduction“ bei akzessorischen Leitungsbahnen [20, 35, 36].

## Klinische Elektrophysiologie

Durrer und Wellens [9] zeigten 1967 bei einem Patienten mit WPW-Syndrom, dass nach einem vorzeitigen elektrischen Impuls mit einem kritischen Kopplungsintervall die akzessorische Bahn antegrad blockierte, und es zu einer Normalisierung des QRS-Komplexes kommt. Der Impuls lief über die normale AV-Leitung und trat retrograd über die akzessorische Bahn wieder zum Vorhof ein („circus movement tachycardia“). 1971 untersuchten Wellens et al. die unterschiedlichen Mechanismen der Tachykardien bei Präexzitationssyndromen [46]. Sie beschrieben die orthodrome und die antidrome Tachykardie bei WPW-Syndrom.

In dieser Zeit war eine medikamentöse Behandlung der Tachykardien bei WPW-Syndrom nur sehr eingeschränkt möglich. Lediglich Procainamid, Quinidin, Verapamil und Propranolol standen zur Verfügung. Bender und Brisse führten schon früh eine Kombinationstherapie mit Verapamil und Quinidin bei supraventrikulären Tachykardien klinisch ein [14]. 1964 konnte Puech erstmalig zeigen, dass eine Injektion von Ajmalin zu einer Normalisierung des QRS-Komplexes führte, wobei es zu einer Blockierung der akzessorischen Leitungsbahn kommt [37]. Wellens et al. [45] untersuchten 1974 die pharmakologischen Effekte von Antiarrhythmika bei Präexzitationssyndromen. Dazu wurden intravenöse Dosen verabreicht und die Effekte auf die Induzierbarkeit einer Tachykardie und der Refraktärität der akzessorischen Bahn untersucht. Damals galt Procainamid als die effektivste Substanz, die Leitungskapazität der Bahn zu verringern. Diese Befunde wurden von Seipel et al. [41] 1974 sowie Wellens et al. [45] zeitgleich publiziert. In der BRD untersuchten Neuss und Schlepper die Eigenschaften ausschließlich retrograd leitender Bahnen und deren Reentry-Mechanismen [32, 33]. Darüber hinaus publizierten die Autoren die Effekte von unterschiedlichen Antiarrhytmika auf akzessorische Leitungsbahnen [31]. Sie untersuchten die Effekte von Verapamil, Aprindin, Ajmalin und Orciprenalin. Neuss verfasste 1976 seine Habilitationsschrift zu „Befunden der His-Bündel-Elektrographie bei Präexzitationssyndromen und paroxysmalen Tachykardien“ in Mannheim [30]. Seipel et al. untersuchten die medikamentösen Effekte von Ajmalin auf die akzessorischen Leitungsbahnen [42]. Ende der 1970er Jahre wurden die Medikamente Propafenon, Flecainid und Sotalol entwickelt und zugelassen. Die Wirksamkeit von Propafenon wurde von Breithardt und Seipel 1984 untersucht [4]. Dazu wurden nach intravenöser Gabe die akuten Effekte analysiert. Propafenon verlängerte das AH-Intervall, die HV-Zeit, QRS-Dauer und die atrialen und ventrikulären Refraktärzeiten. Auch die Refraktärität der akzessorischen Leitungsbahn wurde durch Propafenon verlängert.

Im Jahr 1978 erschien im Thieme Verlag das erste deutschsprachige Elektrophysiologie-Lehrbuch verfasst von Ludger Seipel mit einem Anhang „Funktionsanalyse des Sinusknotens“ unter Mitarbeit von Günter Breithardt [40]. Das Geleitwort verfasste Franz Loogen, Gründer der Kardiologie in Deutschland. Er schreibt darin, „dass bisher zwar einige Berichte von internationalen Symposien zu diesem Thema (Elektrophysiologie) erschienen sind, aber eine zusammenfassende Monografie dagegen noch nicht publiziert wurde. Das vorliegende Buch kann dem Anfänger eine Einführung in die Methoden vermitteln. Darüber hinaus soll es dem Fortgeschrittenen spezielle Probleme näherbringen, wobei die kaum noch zu übersehende Literatur, zusammen mit dem eigenen Befunden, referiert wird.“ Seipel stellt hier, illustriert mit zahlreichen Abbildungen, die Varianten der Präexzitationssyndrome dar und erklärt die Mechanismen der Tachykardien und die möglichen Therapieansätze. Viele der Abbildungen sind original und an keiner Stelle sonst publiziert. Insgesamt diskutiert Seipel die Befunde zu Präaexzitationssyndromen über 30 gedruckte Seiten. Als zu Beginn 1980 Sotalol und Encainide zugelassen wurden, legten Kunze und Kuck 1984 und 1987 Befunde zu elektrophysiologischen Effekten dieser Substanzen bei WPW-Syndrom vor [25, 26].

## Weiterführende elektrophysiologische Diagnostik

Durch die Entwicklung von steuerbaren Elektrodenkathetern und Verminderung des Elektrodenabstands an der Katheterspitze sowie der Entwicklung multipolarer Katheter wurde die Lokalisationsdiagnostik akzessorischer Bahnen deutlich verbessert. Jackman [17] und Kuck [21] konnten zeigen, dass man direkt Potenziale von akzessorischen Bahnen ableiten konnte. Dies war eine entscheidende Voraussetzung für die Entwicklung einer erfolgreichen Katheterablation akzessorischer Leitungsbahnen.

## Nichtpharmakologische Behandlung des WPW-Syndroms

Am 2. Mai 1968 gelang Sealy [8, 39] die erste chirurgische Durchtrennung einer akzessorischen Bahn. Die akzessorische Bahn wurde präoperativ lokalisiert durch die Elektrophysiologen J. P. Boineau und A. G. Wallace. Intraoperativ wurde die Bahn mittels Mapping identifiziert und operativ erfolgreich durchtrennt. In den nachfolgenden Jahren wurden in Paris (Fontaine, Guiraudon, Frank [13] und Menasché, Coumel, Slama [27]), in Zürich (Kappenberger, Turina [19]) und in Maastricht (Penn, Wellens, Brugada [34]) antitachykarde Operationen bei WPW-Syndrom durchgeführt. Hier kooperierten Herzchirurgen und Elektrophysiologen. In der BRD etablierten sich zwei Zentren: Hannover (Frank, Klein und Trappe [11]) und Düsseldorf (Ostermeyer, Breithardt, Borggrefe [1]). Intraoperativ wurde eine Lokalisationsdiagnostik durchgeführt (Mapping). Wegen der Invasivität einer offenen Herzoperation bei ansonsten herzgesunden Patienten, wurde jedoch bald nach Alternativen zur chirurgischen Therapie gesucht. Nach der Einführung der Gleichstromkatheterablationstechnik zur Unterbrechung der AV-Überleitung konnten Gallagher et al. im *New England Journal of Medicine* 1982 [12] und zeitgleich Scheinman et al. in *JAMA* 1982 [38] ihre ersten Ergebnisse zur Gleichstromablationstechnik zur Unterbrechung der normalen AV-Leitung publizieren. 1984 wendete Fisher [10] die DC-Ablationstechnik bei einem Patienten mit linksgelegener akzessorischer Leitungsbahn im Koronarsinus (CS) an. Aber wegen Perforationsgefahr und Tamponade wurde diese Anwendung verlassen. Im Jahr 1984 führten Morady und Scheinman [29] eine DC-Ablation bei einer posteroseptalen Bahn durch. Hierbei lag die Spitze des Ablationskatheters vor der CS-Einmündung. Etwa 65 % der Behandlungen waren erfolgreich. Warin und Haissaguerre [44] konnten 1989 zeigen, dass eine DC-Ablation bei 63 von 70 Patienten erfolgreich war. Die akzessorischen Bahnen waren sowohl rechts- als auch linksseitig lokalisiert. Linksseitige Bahnen wurden über das offene Foramen ovale oder über transseptalen Weg erreicht. Zwei Patienten erlitten einen totalen AV-Block.

**Figure 1:**
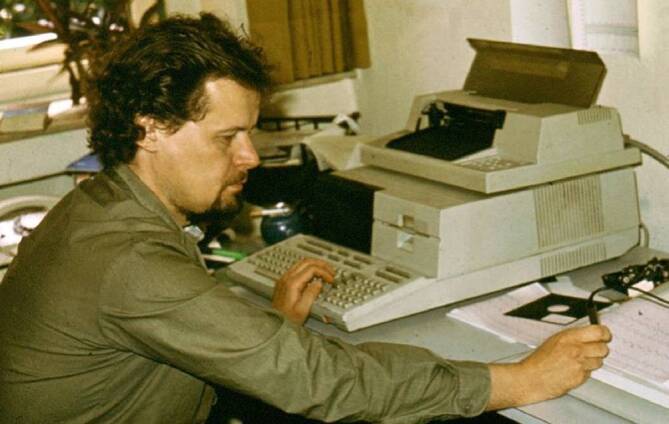

**Figure 2:**
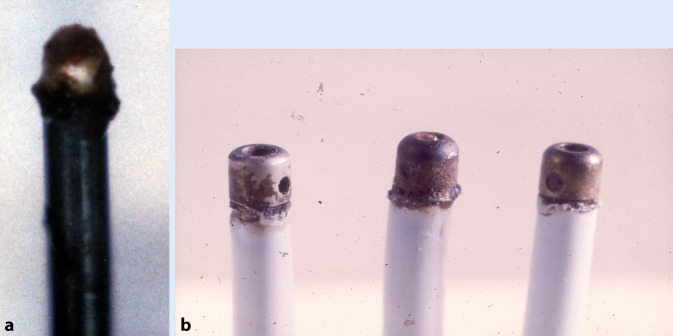

Die DC-Gleichstromablation war jedoch mit signifikanten Komplikationen assoziiert: akute Blutdruckabfälle, Auslösung von Rhythmusstörungen, kardiogener Schock, tödliche Tamponade, Ventrikelruptur und im Verlauf Thromboseformationen im Bereich der Verödungsstellen. Da dieses Verfahren zudem eine Intubationsnarkose erforderte, band es zusätzliches Personal (Kardioanästhesie). Des Weiteren war unter Narkose die hämodynamische Toleranz bei Tachykardie eingeschränkt und zum Teil ließen sich keine Tachykardien mehr unter Narkose induzieren, was eine Lokalisation diagnostisch erschwerte. Deshalb wurde weiter nach Alternativen geforscht. Bereits 1983 versuchte A. Laucevicius aus Vilnius, Litauen (Abb. 1), als Gastarzt an der Universität Düsseldorf, Kardiologie (Prof. Loogen) Ablationen experimentell mit konventionellen Diathermiegeräten durchzuführen. Bei den Ex-vivo-Experimenten wurde ein konventioneller Elektrodenkatheter mit dem Elektrokauter verbunden. Aufgrund der hohen Stromdichte kam es jedoch zu Karbonisationen der Elektrodenspitzen und Einschmelzung der Elektrodenisolation (Abb. 2).

**Figure 3:**
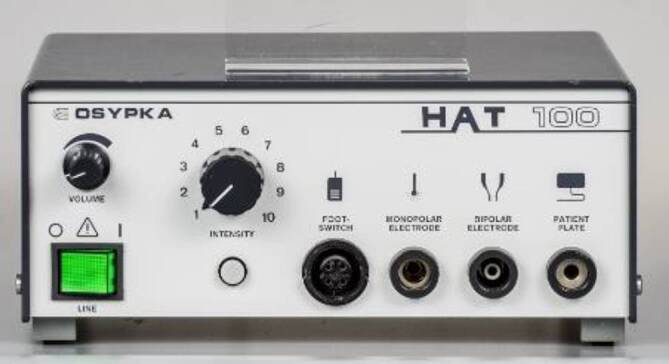

**Figure 4:**
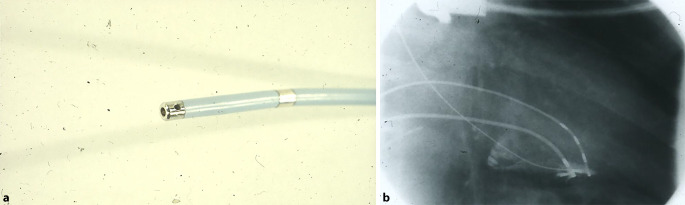

**Figure 5:**
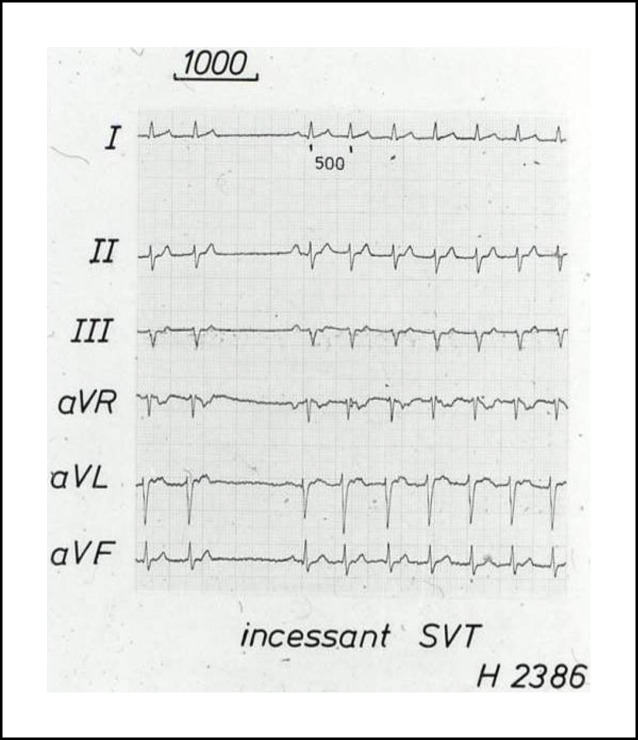

**Figure 6:**
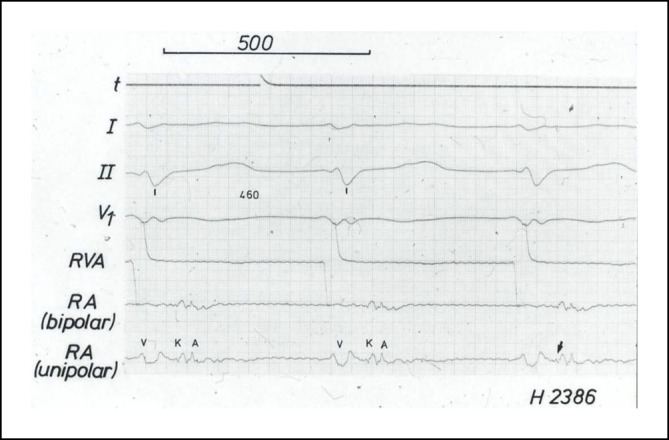

Im Jahr 1985 hat Osypka, ein Ingenieur, interventionell tätige Elektrophysiologen in das Airport Center am Frankfurter Flughafen eingeladen. Unter anderem waren anwesend: Borggrefe, Breithardt, Hoffmann, Kuck, Oeff und Steinbeck. Osypka stellte den Hochfrequenzgenerator HAT 100 (Abb. 3) vor und demonstrierte an in Kochsalz ausgestellten Schnitzeln, wie kleine, umschriebene Läsionen mittels Hochfrequenzstrom induziert werden konnten. Die indifferente Elektrode lag unter der Glasschale, und es wurden Elektrodenkatheter mit einer 2‑mm-Spitze an den Generator angeschlossen. Alle Eingeladenen fuhren sehr beeindruckt wieder zurück an ihre Klinik. In Düsseldorf machten wir uns nach Erhalt eines Hochfrequenzstromgenerators gleich an die Arbeit und testeten das Ablationspotenzial ex vivo an Schweineherzen. Hier brachte sich Thomas Budde sehr ein [5]. Er führte systematische Untersuchungen zur Dosis-Wirkungs-Beziehung der Läsionsgröße durch (Abb. 4a, b). In Abhängigkeit von der gewählten Energiedosis (2,5–50 W) und Dauer der Ablation (10, 30, 60 s) ergaben sich Koagulationszonen von 1,8–8,9 mm Durchmesser und 0,7–4,8 mm Tiefe [3]. Am 18.07.1986 setzte das Team von G. Breithardt und M. Borggrefe sowie Th. Budde und A. Podczeck (Operateur G. Breithardt) dann das Verfahren zur Unterbrechung einer normalen AV-Überleitung bei einer Patientin mit therapierefraktären supraventrikulären Tachykardien (sog. Bachmann-Bündel-Tachykardie) ein. Die Patientin war zuvor mittels DC-Ablationstechnik erfolglos behandelt worden. Zunächst wurde versucht, die atriale Tachykardie im Vorhof zu veröden, was nicht gelang. Dann folgte die Entscheidung zur Durchtrennung der AV-Leitung durch Hochfrequenzstrom. Nach der ersten Applikation trat ein AV-Block I° ein. Nach einer weiteren Verödung folgte ein kompletter AV-Block. Der Ersatzrhythmus zeigte einen schmalen Komplex (QRS-Dauer 70 ms). Nachfolgend erfolgte eine Schrittmacherimplantation. 1987 wurde dieser Fall von Budde et al. publiziert [6]. In der zweiten Jahreshälfte 1986 wurden von Breithardt und Borggrefe 12 Patienten mit einer rechtsgelegenen akzessorischen Bahn mittels RF-Ablation behandelt. In 9 Fällen war die Behandlung erfolgreich und bei einem Patienten konnte die Refraktärzeit der Bahn verlängert werden. 1987 publizierte Borggrefe die erste Hochfrequenzstromablation einer akzessorischen Leitungsbahn (Abb. 5, 6, 7 und 8; [2]). Es war die 10. Applikation der RF-Ablation; die Motivation, diesen Fall zu publizieren, bestand in den interessanten elektrophysiologischen Befunden im Rahmen der Lokalisationsdiagnostik. Es handelte sich um eine ausschließlich retrograd leitende, rechtsseitig gelegene akzessorische Leitungsbahn, die unter fixfrequenter ventrikulärer Stimulation 2:1 retrograd blockierte. Dabei sah man lokal ein ventrikuläres Signal (V), ein Signal der akzessorischen Leitungsbahn (AP), gefolgt vom Atrium (A). Bei Blockierung erkannte man V‑AP-kein A, so dass wir postulierten, dass bei einer Blockierung distal des His, das lokale Elektrogramm die „Schwachstelle“ der retrograden Bahn darstellte. Bei Abgabe eines RF-Impulses kam es zur erfolgreichen Unterbrechung der Leitungsbahn. In der Initialphase dieses neuen Therapieprinzips traute man sich nicht, das Verfahren auf der arteriellen Seite anzuwenden. Einerseits befürchtete man potenzielle Perforationen und andererseits bestand die Gefahr einer thermischen Koronarläsion insbesondere bei Applikationen am AV-Ring. Unsere Arbeitsgruppe führte daher zunächst nur Hochfrequenzstromablationen bei rechts gelegenen Bahnen aus. Experimentell konnte gezeigt werden, dass eine Ablation bipolar im CS und unterhalb der Mitralklappe durchgeführt werden kann. 1988 gelang es Kuck, mittels bipolarer Ablation eine links gelegene Bahn zu modifizieren [23]. Im Jahr darauf wandte Kuck die bipolare Ablationstechnik bei einem Patienten mit einer links gelegenen Bahn an [22]. Der RF-Impuls wurde zwischen einem im CS gelegenen Katheter und einer Elektrode im Bereich des Mitralklappenanulus bipolar abgegeben. Die Behandlung war erfolgreich. Alle bis dahin durchgeführten Hochfrequenzstromablationen wurden mit nichtsteuerbaren Sonden und einer 2‑mm-Elektrodenspitze durchgeführt (Abb. 9). Die Erfolgsrate lag dabei um die 50 % (Borggrefe, Vortrag bei der „American Heart Association“ 1987).

**Figure 7:**
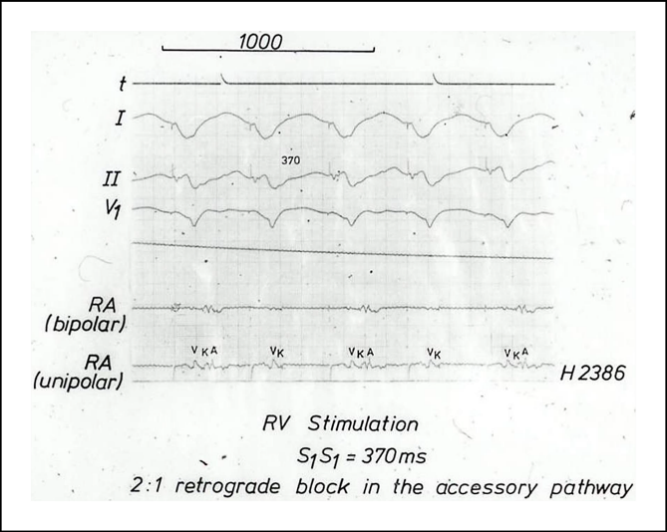

**Figure 8:**
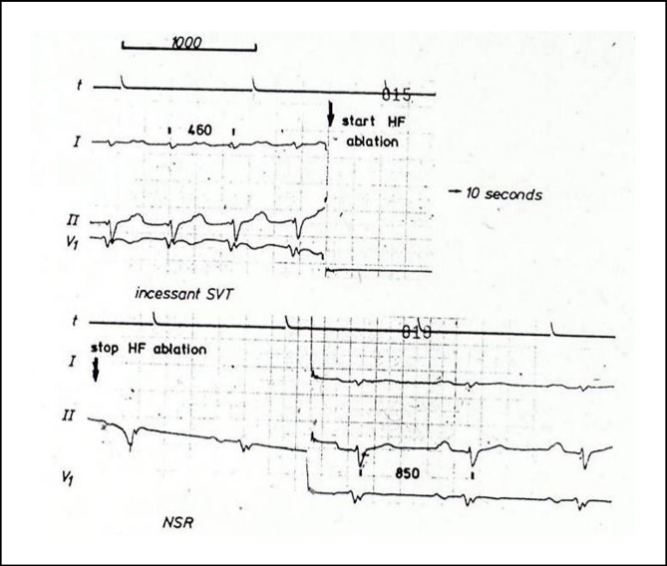

**Figure 9:**
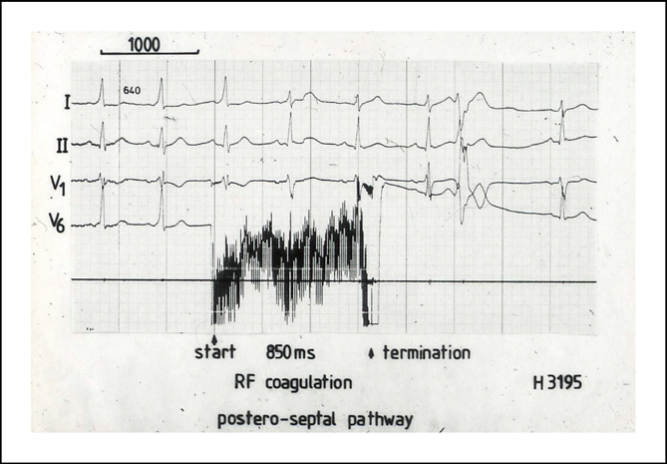

**Figure 10:**
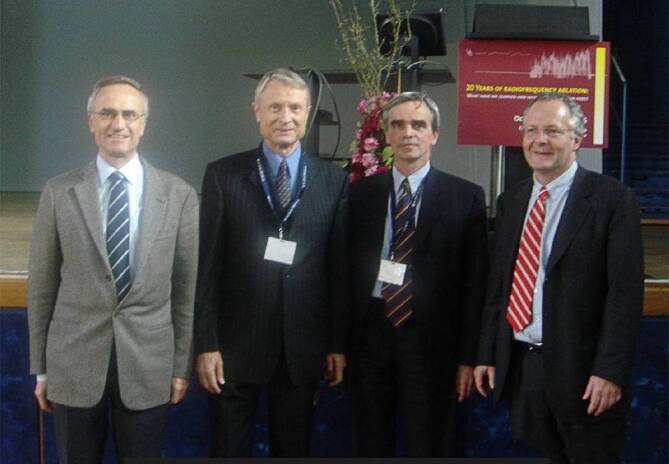

Mit der Einführung einer 4‑mm-Elektrodenspitze und steuerbaren Sonden konnten die Erfolgsraten deutlich gesteigert werden. 1991 publizierten Jackman et al. [18] im *New England Journal *ihre ersten Ergebnisse und im gleichen Jahr Kuck et al. [24] in *Lancet* mit der 4‑mm-Spitzenelektrode. Die Erfolgsraten lagen dabei bei über 90 % mit niedrigen Komplikationsraten. Heute ist die interventionelle Behandlung von akzessorischen Leitungsbahnen ein Routineeingriff geworden (Abb. 10). Allerdings muss man feststellen, dass sich nur noch wenige Patienten mit symptomatischem WPW-Syndrom vorstellen, eine „ausgerottete Spezies“, wie ein amerikanischer Elektrophysiologe feststellte.

Interessant ist, dass bei der Rückschau in der Entwicklung nichtpharmakologischer Therapieverfahren initial bei der chirurgischen Behandlung des WPW-Syndroms 10–20 % der Patienten multiple akzessorische Bahnen aufwiesen. Das Gleiche wurde auch bei der DC-Ablationstechnik beschrieben. Mit der Einführung der RF-Ablationstechnik, verbesserter Lokalisationstechnik und zunehmender Erfahrung, lagen multiple Leitungsbahnen bei nur < 5 % der Patienten vor. Vielleicht war die Annahme multipler Bahnen auch ein Entschuldigungsversuch für eine nichterfolgreiche Ablation in der ersten Zeit.

Über Kongresse, Einladungen zu Vorträgen in elektrophysiologischen Zentren, Durchführung von Ablationen an anderen Standorten von China bis USA, Besuche ausländischer und inländischer Ärzte zum Erlernen der Ablationstechnik ist ein großer Zusammenhalt in der elektrophysiologischen Gesellschaft entstanden. Langjährige Freundschaften haben sich entwickelt. Gemeinsame Essen hatten sich etabliert. Man hat sich gegenseitig wertgeschätzt. Etwas von diesem wünsche ich den interventionell tätigen Elektrophysiologen sich in der Zukunft wieder zu erarbeiten.

Einst beschrieb Francis Marchlinski in einer Übersichtsarbeit invasive Elektrophysiologen als „… learning while burning“ [7]. Er beklagte, dass durch die Einführung von Ablationstechniken das Interesse an Arrhythmiemechanismen verlorengehe. Ich habe beobachtet, dass an vielen nichtuniversitären und universitären Krankenhäusern Elektrophysiologen arbeiten, die zurückgezogen in elektrophysiologischen Labors invasiv tätig sind. „They become lonely while burning“ (Zitat Borggrefe).
